# Research progress on the role of biomechanical clues in the progression of lung cancer

**DOI:** 10.3389/fcell.2025.1581831

**Published:** 2025-10-08

**Authors:** Fengying Gong, Qiang Zhang, Qin Fan, Rongmei Qu, Congrong Wang, Ting Yang, Tingyu Fan, Ying Lv, Jingxing Dai

**Affiliations:** ^1^ Department of Traditional Chinese Medicine, Nanfang Hospital of Southern Medical University, Guangzhou, China; ^2^ The Second Clinical College of Guangzhou University of Chinese Medicine, Guangzhou, China; ^3^ School of Traditional Chinese Medicine, Southern Medical University, Guangzhou, China; ^4^ Guangdong Provincial Key Laboratory of Medical Biomechanics and Guangdong Engineering Research Center for Translation of Medical 3D Printing Application and National Key Discipline of Human Anatomy, School of Basic Medical Sciences, Southern Medical University, Guangzhou, China; ^5^ Department of Laboratory Medicine, Nanfang Hospital of Southern Medical University, Guangzhou, China; ^6^ Department of Oncology, Guangzhou Fuda Cancer Hospital, Guangzhou, China

**Keywords:** biomechanical clues, lung cancer, tumor microenvironment, extracellular matrix, stiffness, mechanical stress, stretching

## Abstract

Cells in mammals perceive and react to the mechanical properties of their surrounding environment. Disease progression is frequently linked to dynamic changes in cellular and tissue mechanics. Mechanical responses have been investigated in a broad range of pathological states, notably viral and bacterial infections, inflammation, cystic fibrosis, and tumorigenesis. The lung is an inherently mechanosensitive organ. As such, it is subjected to tremendous mechanical forces. Evidence suggests that lung tumors are subjected to and react to active and passive forces that are critical for their initiation, differentiation, migration, and effector functions, as well as those of their extracellular matrix. This review discusses the latest advances in the investigation of the mechanics of lung cancer cells, focusing on the effects of mechanical signals from tumor microenvironment on tumor cell metabolism and tumor aggressiveness. Investigating the biological impacts of stress and stiffness alterations in lung cancer cells and their associated extracellular matrix can enhance our understanding the pathogenesis of lung cancer and offer novel insights for future therapeutic strategies.

## 1 Introduction

Respiration maintains metabolism via gas exchange (external, transport, and internal). The lung is mechanosensitive, with alveoli stretching up to 12%. Mechanical stress affects cell growth and repair—excessive strain causes apoptosis and delays healing. These changes are mediated by ECM alterations, influencing disease development. After lung injury, epithelial cells repair under mechanical strain. Cancer cells demonstrated distinct mechanical properties, including cellular stiffness and elasticity, which were intricately linked to cell migration, metastasis, and epithelial-mesenchymal transition (EMT) processes (Cross et al., 2007). The physical properties of lung cancer cells, especially stiffness, represent an important aspect of their biological behavior. Studies have shown that lung cancer cells have lower mechanical strength than normal lung cells. Lung cancer cells exhibit increased deformability, which renders them more prone to invading adjacent tissues and blood vessels (Wohl et al., 2023). This review discusses the latest advances in the investigation of the mechanics of lung cancer cells, focusing on the effects of mechanical signals from tumor microenvironment on tumor cell metabolism and tumor aggressiveness. Investigating the biological impacts of stress and stiffness alterations in lung cancer cells and their associated extracellular matrix can enhance our understanding the pathogenesis of lung cancer and offer novel insights for future therapeutic strategies.

## 2 Physiological biomechanics of the lungs

Respiration is essential for the maintenance of normal metabolism and life activities. The primary function of the respiratory system is to facilitate gas exchange. The respiratory process consists of three segments, namely, external respiration (or pulmonary respiration), gas transport, and internal respiration (or tissue respiration) (Hsia et al., 2016; Novak et al., 2021). The lung is a naturally mechanosensitive organ and experiences continuous mechanical stimuli resulting from respiratory movements. Under physiological conditions, the alveolar region is subjected to linear strains of up to 12% (Waters et al., 2012). Mechanical stress exerts significant influence on cellular elongation, multiplication, mitosis, and other biological processes (Wang et al., 2016). Studies have shown that scarred tissue is less flexible than healthy tissue and experiences compensatory strain, resulting in greater mechanical tension. High levels of mechanical stretch were reported to induce apoptosis in rat alveolar epithelial cells, delay wound repair processes, as well as decreases the migration of 16HBE14o−cells (Desai et al., 2008). Furthermore, periodic mechanical stretching was shown to slow wound healing in primary human alveolar epithelial cells (Ito et al., 2014). The majority of biomechanical alterations were primarily mediated by modifications in the composition of the lung extracellular matrix (ECM) (Burgess and Harmsen, 2022; Burgstaller et al., 2017; Suki et al., 2022). For instance, alterations in ECM rigidity had been shown to influence alveolar epithelial cell behavior via mechanotransduction and promote disease development (Ulldemolins et al., 2024). Following acute lung injury, alveolar epithelial cells undergo repair on substrates subjected to cyclic mechanical deformation.

## 3 Biomechanical properties of lung cancer cells

### 3.1 Mechanical stiffness and deformability

**FIGURE 1:**
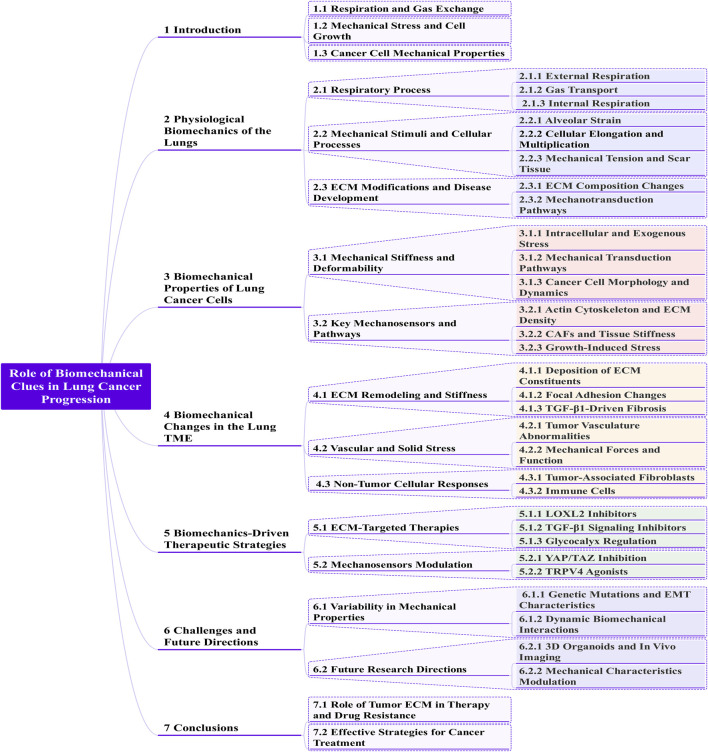
A Mermaid mind map representation of extrinsic forces modulate lung cancer.

Cells in the organism are subjected to various forms of mechanical stimulation, including fluid shear force, fluid pressure, tensile and compressive forces of the surrounding matrix, etc. According to the source of mechanical stress, it can be divided into intracellular stress mediated by the mechanical characteristics of ECM, and exogenous stress such as solid/liquid pressure, tensile force, and fluid shear stress (Figure 1) (Doyle et al., 2022; Guo et al., 2022; Liebman et al., 2020). Like normal cells, tumor cells can also sense the mechanical changes in tumor microenvironment and transform them into signaling pathways through mechanical transduction pathways, thereby affecting their abilities such as proliferation, migration, and invasion (Shu et al., 2024; Zhang et al., 2025). Under limited space and resource conditions, excessive proliferation of tumor cells means that they must withstand more mechanical or physical pressure. Tumor cells are exposed to various mechanical forces, including intercellular tension, interstitial fluid pressure, compressive stress, and fluid shear force, etc (Shu et al., 2024; Zhang et al., 2025).

Cancer cells demonstrated distinct mechanical properties, including cellular stiffness and elasticity, which were intricately linked to cell migration, metastasis, and epithelial-mesenchymal transition (EMT) processes. Compared to normal mesothelial cells in the body fluids, cancer cells from lung, breast, and pancreatic cancer patients had a lower Young’s moduli, indicative of lower stiffness (equivalent to less elasticity) (Cross et al., 2007). Lung cells are routinely exposed to cyclical mechanical stresses throughout the respiratory process. In A549 cells, sustained contraction under physiological conditions induces cellular rearrangements and changes in mitochondrial length (Wang et al., 2020). McAdams et al. (2006), demonstrated that the amplitude of cell membrane vibration and the dissipation of mechanical energy are significantly greater in malignant cells compared to benign cells.

The physical properties of lung cancer cells, especially stiffness, represent an important aspect of their biological behavior. Studies have shown that lung cancer cells have lower mechanical strength than normal lung cells. Lung cancer cells exhibit increased deformability, which renders them more prone to invading adjacent tissues and blood vessels (Wohl et al., 2023). The mechanical features of lung tumor cells, including morphology, rigidity, and intracellular dynamics, play a pivotal role in determining their physiological and pathological behaviors. It has been found that the amplitude of cell membrane vibration and the dissipation of mechanical energy are significantly greater in malignant cells compared to benign cells (Wohl et al., 2023). Low-stiffness lung cancer cells can better adapt to and traverse mechanically stressful environments, which facilitates their growth and metastasis.

### 3.2 Key mechanosensors and pathways

The mechanical stiffness of lung cancer cells is modulated by both the intracellular microstructure and the ECM. The intracellular microstructure, including the organization and density of the actin cytoskeleton, as well as the density and degree of crosslinking of collagen and other components in the ECM, substantially influence the mechanical properties, particularly the stiffness, of lung cancer cells.

Lower tumor tissue stiffness accelerated the proliferation of tumor cells and facilitated their invasion of surrounding healthy tissue, while increased stiffness led to an invasive phenotype in tumor cells (Chen et al., 2020). The interplay between tumors and their mechanical microenvironment spans multiple spatial scales. At the cellular level, cancer-associated fibroblasts (CAFs) become activated during tumor progression, promoting stromal desmoplasia and increasing tissue stiffness (Ishihara et al., 2017; Jiang et al., 2022; Pankova et al., 2016). Additionally, tumor cells and CAFs generate growth-induced stress through collagen stretching and cell-ECM interactions during migration (Butcher et al., 2009; Mierke, 2019). At the tissue level, however, this stress is constrained by the surrounding host tissue, enabling tumor expansion and invasion through mechanical deformation of adjacent structures. A deeper understanding of these multiscale mechanisms—through combined experimental and theoretical approaches—is essential to elucidate the understudied role of biomechanical factors in tumor progression and treatment.

Cyclic stretching in A549 cells led to cytoskeleton enrichment and mitochondrial reorganization, thereby increasing the invasiveness of lung adenocarcinoma (Wang et al., 2020). In lung cancer patients, mechanical ventilation was associated with an increased abundance of PCSK9 and a higher proportion of metastases, consequently leading to reduced survival rates (López-Alonso et al., 2022). The activation of receptor tyrosine kinase has been found to promote cell softening and motility and accelerate the progression of malignant disease (Iida et al., 2017). Furthermore, the interaction between perfluorooctanoic acid and transmembrane integrins reportedly triggered alterations in cellular mechanical properties, leading to cytoskeletal reorganization and to trigger activation of the intracellular focal adhesion kinase (FAK)-phosphoinositide 3-kinase- Protein Kinase B pathway. This cascade of events ultimately promoted the migration and invasion of lung cancer cells (Zwaans et al., 2022). Cells sustain external forces by regulating cytoskeletal stiffness, while microtubules act as compression-bearing elements. One study demonstrated that mechanically induced cellular metabolism increases microtubule glutamylation, which promotes metastasis (Torrino et al., 2021).

By interacting with the transcription factor Y-box binding protein 1 (YBX1), LINC00472 downregulates the expression of vimentin, leading to a more dense and organized microfilaments. This results in increased cellular stiffness in A549 cells, thereby inhibiting EMT process and consequently suppressing the invasion and metastasis of lung adenocarcinoma cells (Deng et al., 2020). One study demonstrated that gold nanoparticles can elicit mechanobiological responses in lung cancer cells *in vivo*, largely reducing their migratory potential (Sohrabi Kashani et al., 2022).

Cell hardness changes following cancer cell transformation. TGF-β1-induced epithelial-mesenchymal transition (EMT) can be rapidly detected within seconds during the intravasation and extravasation of cancer cells (Wu et al., 2014). An *in vitro* analysis showed that Lewis lung cancer (LLC) cells acquire stronger cytomechanical properties and movement post-EMT, effects that were subsequently found to be correlated with a decline in body-weight gain and an increase in tumor weight *in vivo* (Wu et al., 2014).

Large tumors exhibit higher mesenchymal stress. It was shown that the mitotic index and immunoreactivity for nuclear transcription factor-kappa B (NF-κB), phosphorylated IκB, and cell cycle protein D1 in the central regions of 28 human lung, colon, head, and neck tumors were significantly higher than those in the corresponding peritumoral tissues. The rapid growth of large, rigid tumors can raise intra-tumor tension and stimulate tumor proliferation via mechanosensitive calcium channels, creating a feedback loop that promotes tumor proliferation. Interfering with this signal may prevent the progression of large, unresectable tumors (Zwaans et al., 2022).

Numerous studies have shown that mechanical force plays an important role in the occurrence and development of lung cancer. The interaction between cells and ECM promotes cells' perception of mechanical forces, which are then converted into biochemical signals, thereby triggering biological reactions. Including FAK (Shikata et al., 2005), mechanosensitive protein YAP/TAZ (Dupont et al., 2011), tension-sensitive ion channels Piezo1/Piezo2 (Douguet and Honore, 2019; Huang et al., 2019), Rho GTPases and force transduction proteins, etc. (Doyle et al., 2022), which further affect the evolution of idiopathic pulmonary fibrosis into lung cancer and promote the proliferation of lung cancer cells.

Short Summary: Tumor cells sense mechanical forces (ECM-mediated stress, fluid pressure, shear stress) and convert them into biochemical signals, influencing proliferation, migration, and invasion. Lung cancer cells exhibit lower stiffness (Young’s modulus) than normal cells, enhancing deformability for metastasis. Reduced stiffness promotes invasion, while increased ECM stiffness drives aggressive phenotypes.

Key factors modulating stiffness:

1. Cytoskeleton and ECM: Actin organization, collagen crosslinking.
2. Mechanical stress: Cyclic stretching enriches cytoskeleton, boosts invasiveness.
3. Signaling pathways: FAK, YAP/TAZ, Piezo channels, and Rho GTPases transduce forces into pro-tumor signals.
4. EMT: softening during EMT aids intravasation/extravasation.

## 4 Biomechanical changes in the lung TME

### 4.1 ECM remodeling and stiffness

The tumor microenvironment (TME) encompassed the local milieu surrounding tumor cells, comprising not only the tumor cells themselves but also supporting stromal cells, ECM, vasculature, immune cells, and a myriad of signaling molecules. Cancer cells proliferated within a mechanistically and chemically heterogeneous microenvironment that evolved dynamically throughout tumor progression (Kraning-Rush et al., 2012). Microenvironmental stiffening exerts a significant influence on the process of tumorigenesis. Filamentous pseudopods were hypothesized to function as cellular mechanosensors, enabling the detection of environmental stiffness. Additionally, whether a filamentous foot extends or retracts is a purely stochastic process that does not depend on basal stiffness. Filamentous pseudopod activity is closely regulated by the strength of cell adhesion (Liou et al., 2014). Elevated solid tissue stiffness exacerbates lung cancer advancement and worsens prognosis. The study demonstrated that rigidity-induced autophagy in stromal cells, including fibroblasts and stellate cells, played a crucial role in promoting the growth of adjacent cancer cells both *in vitro* and *in vivo*. This autophagic process is reliant on integrin αV, which targets tumor-mesenchymal crosstalk. Changes in mechanical tissue properties alone are sufficient to metabolically reprogram stromal cell populations, thereby generating a cancer-supporting metabolic niche. Alterations in tissue mechanics alone could reprogram stromal cells to create a metabolic environment that supports tumor growth (Hupfer et al., 2021).

Pulmonary connective tissue is composed of lung cells and ECM. The characteristics of the ECM are defined by its components, particularly proteoglycans, collagen, and elastin. Ulldemolins et al. (2024), proposed that found that lung stiffness increases with age due to changes in ECM proteins like fibronectin, elastin, and laminin. Lin et al. (2024), tested A549 adenocarcinoma cell spheroids in collagen matrices with varied stiffness and fiber density. They found that compressed collagen inhibited spheroid expansion but did not affect tumor growth. Instead, it increased MMP activity, correlating with reduced matrix stiffness. These findings indicated that fibrous structures counteract matrix stiffness-induced motion (Lin et al., 2024). The basement membrane is an ECM substructure with a width of only 100–400 nm whose biomechanical properties are critical for tumor progression and metastasis. Alveolar function is dependent on the mechanical strength of the ECM and its responses to external forces. The basement membrane is stiffer than the adjacent cellular layer (Hartmann et al., 2024; Sicard et al., 2017). The components of the ECM of lung cancer include CAFs, vascular networks, endothelial cells, mesenchymal stem cells, immune cells, and soluble substances. ECM stiffness can regulate cancer cell growth and phenotype (Tilghman et al., 2010). *In vivo*, cells exerted pulling forces on the surrounding three-dimensional (3D) extracellular matrix and adjacent cells. These traction forces may increase stiffness and remodel the matrix, which, in turn, affects cellular function. This dynamic interplay mediates tumor development (Emon et al., 2021).

Fibroblasts, human colon cancer cells (FET), and A549 cells demonstrate marked force variations in the 3D ECM. In a FET/CAF co-culture model, which mimics the TME, tissue stiffness tripled within 24 h (Emon et al., 2021). Mechanical signals emitted by the ECM can affect the density distribution of cells (Baday et al., 2019). It was noted that cells seeded in a matrix with poor collagen density have enhanced migratory potential. The cells migrate away from their native clusters, favoring the generation of microstructures (Gonçalves and Garcia-Aznar, 2021). A study found that changing matrix hardness enhanced the proliferation and aggressiveness of A549 cells in 3D culture (Alonso-Nocelo et al., 2018). Additionally, substrate hardness was observed to modulate the migration of lung cancer cells via focal adhesion signaling, but not EMT signaling (Shukla et al., 2016). The discrepancy in these findings concerning ECM stiffness’s impact on tumor cell EMT likely arises from the different culture systems used: 3D culture versus two-dimensional culture.

#### 4.1.1 Deposition of ECM constituents

Stromal sclerosis due to ECM deposition is closely associated with tumor progression. The spindle pole body component 25 homolog, upregulated by ECM stiffening, was crucial for lung cancer cell proliferation (Jeong et al., 2018). Mechanical signals originating from ECM could induce the transition to a malignant phenotype. Metastasis, responsible for more than 90% of cancer deaths, was regulated by intracellular forces. Cellular contractility determined the matrix rigidity needed for optimal function and influenced metastatic cancer cell localization during tissue implantation (McGrail et al., 2015).

##### 4.1.1.1 Collagen

Epithelial tumor metastasis was driven by collagen cross-links, which made the surrounding tissue stiffer and helped tumor cells become more invasive. Compressed collagen constructs inhibited the expansion of A549 adenocarcinoma cell spheroids, activated MMP activity, and reduced hardness without affecting the proliferative capacity of tumor cells (Lin et al., 2024). It was found that both CAFs and LH2 induce collagen cross-linking switches in tumor stroma and enhance the metastatic properties of tumor cells (Chen et al., 2015; Pankova et al., 2016). Type IV collagen has been implicated in the regulation of tumor cell stiffness and migration by activating integrin signaling pathways (Chen et al., 2014). Integrin α11β1, a receptor for fibrillar collagen, is hyper-expressed during the differentiation of fibroblasts into CAFs. Integrin α11β1 facilitates NSCLC tumorigenesis and metastasis by mediating collagen reorganization and modulating stromal stiffness within the tumor microenvironment (Navab et al., 2016). Studies have demonstrated that the mechanics of the body undergo marked changes under pathological conditions, such as abnormal type I collagen cross-linking and deposition in the microenvironment of organ fibrosis or solid tumors. These changes lead to a significant increase in elastic modulus of microenvironment, and an increase in the overall stiffness of the ECM. These modifications increased the elastic modulus of the microenvironment, enhancing the stiffness of ECM (Dongre and Weinberg, 2019; Tian et al., 2022).

##### 4.1.1.2 Epithelial-mesenchymal transition

Matrix stiffness increased due to ECM deposition surrounding cancer cells were concurrently linked with epithelial-mesenchymal transition. Overexpression of the EMT marker gene p300 during stromal stiffening and/or subsequent DDR2 upregulation via c-Myb-mediated acetylation may drive EMT gene activation and increased lung cancer cell invasiveness (Kim et al., 2017). Moreover, the stiffness of the physiological matrix had been shown to influence both the quantity and protein content of small extracellular vesicles secreted by cancer cells, which subsequently facilitated the metastasis of cancer cells (Sneider et al., 2024). ECM stiffness contributes significantly to the regulation of cellular behavior; however, its importance in tumor invasion remains incompletely understood.

#### 4.1.2 Focal adhesion changes and responses

Focal adhesions (FAs) are mechanosensory structures that convert physical stimuli into chemical signals that direct cell migration. The inhibition of neuregulin expression in normal and tumor cells resulted in a reduction in FA volume and fluorescence intensity but did not affect cell migration into the wound (Li et al., 2023). Evidence showed that zyxin acted as a critical mechanotransducter, essential for regulating gene expression (Sun et al., 2021). Zyxin enhances actin polymerization via enabled (Ena)/vasodilator-stimulated phosphoprotein proteins in response to mechanical tension, thereby promoting stress fiber remodeling and repair. Zyxin relocated from FA to the nucleus in response to mechanical stretch, modulating gene transcription by interacting with transcription factors, including nuclear matrix protein 4 (Wang et al., 2019a).

#### 4.1.3 TGF-β1-driven fibrosis and altered cellular mechanical properties in the tumor microenvironment

TGF-β1 signaling promoted collagen accumulation and fibrosis, exhibited anti-inflammatory effects, and inhibited epithelial cell proliferation. Enhanced cellular stiffness represented a prominent mechanical characteristic associated with TGF-β1-induced epithelial-mesenchymal transition (Thoelking et al., 2010). TGF-β1-induced cytoskeletal remodeling during cancer cell transformation mediates changes in NSCLC cell stiffness (Gladilin et al., 2019). NF-κB, a major modulator of the immune response and cancer progression, is also responsive to substrate hardness. Substrate stiffness modulated NF-κB activeness through actomyosin contraction, causing H1299 lung adenocarcinoma cells on rigid substrates to appear more dispersed (Ishihara et al., 2013).

### 4.2 Vascular and solid stress

The tumor vasculature is characterized by morphological abnormalities and hyperpermeability, the extent of which can influence tumor aggressiveness and drug delivery to the tumor. Tumors disrupted vascular homeostasis, causing abnormal blood vessel growth and excessive accumulation of fibrillar collagen with varied stiffness (Zeltz et al., 2020). The sclerosing of stroma surrounding solid tumors increases vascular permeability. Matrix stiffness regulated endothelial barrier integrity by activating focal adhesion kinase (FAK) (Wang et al., 2019b). Transient receptor potential vanilloid 4 (TRPV4) channels acted as mechanosensors in endothelial cells, regulating reorientation from cyclic strain and nitric oxide production from shear stress. These channels preserved tumor vascular integrity by sustaining VE-cadherin expression at cellular junctions (Cappelli et al., 2019). TRPV4 regulated tumor vasculature formation and maturation by modulating mechanosensitivity of tumor endothelial cell (Adapala et al., 2016). Similarly, TRPV4 regulates endogenous angiogenesis by modulating endothelial cell mechanosensitivity via the Rho/Rho kinase pathway (Thoppil et al., 2016).

Tumors are complex and heterogeneous tissues comprising not only neoplastic cells but also a diverse microenvironment, including fibroblasts, immune cells, and endothelial cells. These cells, in conjunction with a specialized ECM, established a conducive microenvironment that facilitates tumor progression (Shukla et al., 2016). Mechanical forces in the extracellular microenvironment of alveolar epithelial cells could mediate their function. The glycocalyx is a thick layer of polysaccharides found on the cell membrane surface that serves as a messenger between cells and their surroundings.

### 4.3 Non-tumor cellular responses

#### 4.3.1 Tumor-associated fibroblasts

ECM homeostasis disturbances led to new paracrine signaling, cell-cell communication, and cell-ECM interactions, with critical implications for tumor cell proliferation, invasion, metastasis, immunosuppression, and/or drug resistance. CAFs, key producers of ECM and paracrine signals, played a crucial role in these processes (Zeltz et al., 2020). Activated fibroblasts comprise a category of stromal cells engaged in cancer progression, with EMT serving as one of their sources (Mina et al., 2017). Sclerosing lung cancer stroma and integrin β1 drove subtype-specific fibroblast accumulation. Squamous cell carcinoma (SCC)-associated tumor fibroblasts (SCC-TAFs) showed exhibited significantly elevated expression levels of extracellular regulated protein kinases 1/2 (phosphorylated T202/Y204), FAK (phosphorylated Y397), and integrin β1 compared to adenocarcinoma-associated tumor fibroblasts. The findings of this study supported that the treatments aimed at restoring pristine lung elasticity and/or integrin β1-dependent mechanoregulation may prove effective in combating SCC-TAFs (Puig et al., 2015).

CAFs expressing CD248 were shown to induce NSCLC metastasis through the Hippo pathway, which promotes a collagen I environment in the stromal matrix (Wu et al., 2024). CAFs altered collagen cross-linking in the tumor stromal, affecting the invasiveness of lung tumor cells (Pankova et al., 2016). Tumor matrix mechanics activated glycolysis and glutamine metabolism, coordinating amino acid availability to sustain tumor growth and malignancy (Bertero et al., 2019).

#### 4.3.2 Immune cells

The tumor microenvironment comprised the extracellular matrix, stromal cells including CAFs, infiltrating immune cells, and the vascular system (Rahir and Moser, 2012). Within the TME, tumor-associated macrophages (TAMs) could make up to 50% of the tumor mass, drawing significant attention (Sousa et al., 2015). TAMs were key drivers of stromal transformation, tumor cell growth, metastasis, and adaptive immunosuppression in a variety of cancers (Aras and Zaidi, 2017). Infiltrating macrophages secreted cytokines and enzymes that modify the structure of the ECM, promote fibrosis, and increase matrix stiffness (Clarke et al., 2013; Lech and Anders, 2013). TAMs abundance and ECM stiffness collectively induced an aggressive phenotype and facilitated the upregulation of core EMT marker expression. In contrast to M0 macrophages, M2c macrophages along with stromal stiffness significantly contributed to the emergence of a mesenchymal phenotype and promoted metastasis in A549 lung adenocarcinoma cells (Alonso-Nocelo et al., 2018). Macrophage migration capacity also decreased significantly with increasing 3D matrix stiffness (Adlerz et al., 2016). While it was reported that matrix rigidity does not shape the macrophage phenotype (Alonso-Nocelo et al., 2018), it has also been suggested that matrix stiffness alters the adhesion properties of macrophages, and they release pro-inflammatory cytokines when cultivated on solid substrates (Blakney et al., 2012; Fujisaki and Futaki, 2023; Previtera and Sengupta, 2015). Indeed, certain TAM phenotypes secrete MMPs, which can degrade ECM, altering its hardness and components, and thereby enabling cells to disseminate and shed off tumor lesions (Afik et al., 2016; Kessenbrock et al., 2010).

Natural killer (NK) cells eliminated target cells without prior antigen recognition, forming a core part of the innate immune system. Under both physiological and pathological conditions, these cells can experience different mechanical stimuli, such as fluid shear in blood, solid stress in tissues, and target interface tension (Friedman et al., 2021). It has been shown that NK cell infiltration was markedly influenced by alterations in the mechanical features of tumor stroma and target cell (Zhou et al., 2022), while fluid shear enhances NK cell killing of circulating tumor cells via NKG2D-mediated mechanosensing (Hu et al., 2023). In addition, NK cells exhibit substrate stiffness-dependent activation (Mordechay et al., 2021). The findings indicated that mechanical stimulation exerted a significant influence on the modulation of NK cell immune function. Typically, target cell killing by NK cells is based on the formation of stable immune synapses, a process that involves factors such as adhesion molecules and mechanistic events such as cytoskeletal remodeling-mediated immune adhesion (Ben-Shmuel et al., 2021). The formation and cytotoxicity of NK cell immunological synapses are governed by the stress of the target interface (Friedman et al., 2021). Piezo1 mechanosensing regulated NK-cell cytotoxicity and infiltration in 3D matrices (Yanamandra et al., 2024). Additionally, increasing the speed and persistence of NK cells reduced the search time for target cells, resulting in increased killing efficiency (Zhou et al., 2017).

Mechanosensing and mechanotransduction mediated by lymphatic endothelial cells (LECs) served as critical regulators of lymphatic development and function (Bálint and Jakus, 2021; Oliver et al., 2020). Furthermore, LECs possessed the capability to detect and react to alterations in ECM stiffness as well as cell tension and shear stress induced by fluid pressure. These mechanistic events modulate the immunological activity of lymphocytes (Planas-Paz et al., 2014).

## 5 Biomechanics-driven therapeutic strategies

### 5.1 ECM-targeted therapies to lung cancer

Upregulated by increased matrix stiffness, Lysyl oxidase-like 2 (LOXL2) enhanced the expression of CXCL12, the production of MMP9 and fibronectin, and the uptake of bone marrow-derived dendritic cells. These actions collectively facilitated the formation of a pre-metastatic microenvironment (Wu et al., 2018). LOXL2 is a member of the lysyl oxidase family with multifaceted biological functions, primarily involved in extracellular matrix (ECM) remodeling and tumor progression. Its key roles include: ECM remodeling and collagen crosslinking (Neumann et al., 2018; Zhang et al., 2021), promotion of tumor metastasis (Nguyen et al., 2019), transcriptional and epigenetic regulation (Ikenaga et al., 2017), immune modulation in tumor microenvironment (Nguyen et al., 2019), and non-enzymatic roles (Wen et al., 2020). Elevated matrix stiffness influenced tumor cell behavior, modulated gene, and microRNA expression, and promoted invasion and metastasis (Fu et al., 2023). The early expression of endogenous TGF-β1 affected the mechanical properties of tumor cells as well as tumor growth, angiogenesis, and metastasis. Reciprocally, increased cell stiffness and adhesion force can enhance the cell-environment contact and crosstalk (Wu et al., 2014). Meanwhile, Wei et al. (2017), identified trihydroxyphenolic compounds as potent inhibitors of TGF-β1 signaling, Snail1 expression, and collagen deposition in models of pulmonary fibrosis and lung cancer metastasis. Their activity uniquely depends on LOXL2, confining the effects to LOXL2-producing fibroblasts and cancer cells. Mechanistically, these compounds auto-oxidize a LOXL2/3-specific lysine residue (K731), irreversibly inhibiting the enzyme. This reaction also generates a novel metabolite that directly blocks TβRI kinase. This dual inhibition of LOXL2 and TβRI potently blocks pathological collagen accumulation *in vivo* while avoiding the toxicity of broader inhibitors.

The glycocalyx mainly consists of chondroitin sulfate, heparan sulfate, and hyaluronic acid (Kanyo et al., 2020; Martins et al., 2002). Dynamic stretching stresses coupled with variations in mesenchymal stiffness modify the regulation of glycocalyx-related gene expression. When dynamic force was applied, the expressions of heparan sulfate proteoglycans syndecans one and heparan sulfate proteoglycans syndecans 4 were upregulated in A549 cells on soft substrates compared to rigid substrates (Kohon et al., 2023). The increase in matrix stiffness inhibits the expression of glycocalyx in endothelial cells (Mahmoud et al., 2021). The glycocalyx is far from an inert coating. It is a dynamic, multifunctional barrier that contributes directly to the major challenges in lung cancer treatment: 1) Chemotherapy/Targeted Therapy Resistance: by blocking drug delivery and activating survival pathways; 2) Immunotherapy Resistance: by physically shielding cancer cells from immune recognition. 3) Metastasis: by aiding cancer cell survival and spread. Therefore, elaborating on the glycocalyx is crucial because future combination therapies will likely need to include agents that modulate or disrupt the glycocalyx. This approach could “prime” the tumor, making it more vulnerable to subsequent standard treatments like chemotherapy, targeted drugs, and immunotherapy, ultimately improving outcomes for lung cancer patients. Alveolar epithelial cells experienced continuous cyclic stretch, and fibrotic or cancerous lungs show increased ECM stiffness. In lung cancer, the TME was reshaped to a genic phenotype (Lee et al., 2014). Fibroblasts, fibrocytes (Saijo et al., 2018; van Deventer et al., 2013; Zhang et al., 2013), and mesenchymal stem cells (Quante et al., 2011) have an enhanced role in the TME, leading to the hardening of the mesenchymal ECM (Kuhn et al., 1991; Zhang et al., 1994), on which epithelial cells depend.

### 5.2 Mechanosensors modulation

#### 5.2.1 YAP/TAZ inhibition

Targeting mechanosensors (e.g., YBX1, UBTD1) or nanoparticles may inhibit metastasis. Alpha-mangostin and UBTD1 modulation (via RhoA/YAP) offer potential strategies. Large tumors elevate mechanical stress, activating proliferation via calcium channels—a target for unresectable cancers. Alpha-mangostin, a xanthone compound derived from Garcinia mangostana (mangosteen), exhibits diverse pharmacological activities including antitumor, antioxidant, anti-inflammatory, and antibacterial effects (Sakpakdeejaroen et al., 2022). Alpha-mangostin is a candidate anti-cancer compound with the capacity to alter the microstructure of the actin cytoskeleton and decrease the physical stiffness of lung cancer cells (Phan et al., 2020). UBTD1 was a highly conserved ubiquitin-like protein throughout evolution. Its expression was downregulated in gastric cancer cells and tissues, and its levels correlated with patient survival rates. These findings suggested that UBTD1 may function as a potential tumor suppressor (Zhang et al., 2015). Furthermore, UBTD1 exhibited high expression levels in cells and tissues subjected to continuous or intermittent mechanical stresses, such as skin, heart, lungs, and thyroid (Uhlen et al., 2015; Uhlen et al., 2010). A decline in UBTD1 expression was found to induce RhoA activation, increase cellular adhesion, and activate YAP-associated protein signaling via ROCK2, thus promoting cancer cell invasiveness (Torrino et al., 2019). Yang et al. (2020), showed that stromal stiffness promotes liver cancer progression through a CXCR4-mediated mechanism in which UBTD1 downregulation leads to YAP pathway activation.

#### 5.2.2 TRPV4 agonists to normalize vasculature

TRPV4 channels are key regulators of tumor angiogenesis and that TRPV4 upregulation and activity are inhibited in tumor vascular endothelial cells. These results further indicate that the activation or restoration of TRPV4 expression induces vascular normalization and improves cancer therapy.

## 6 Challenges and future directions

Variability in mechanical properties across lung cancer subtypes. Malignant conversion, driven by genetic mutations in cells, was also influenced by modifications in cellular properties and EMT characteristics, including changes in stiffness and adhesion. When transformed cells underwent malignant progression, they interacted with their microenvironment through direct physical contact and the application of mechanical forces. Mechanical stresses may alter cell and microenvironment properties, influencing cell fate.

The tumor microenvironment involved dynamic biomechanical interactions between the ECM’s physical properties and tumor progression. Alterations in stress and stiffness properties within the ECM of lung cancer cells were crucial to their biological behavior. The mechanical properties of the tumor microenvironment may vary significantly among different types of lung cancer and even among same type patients. This will also be one of the future research directions. Consequently, a comprehensive and rigorous investigation into this field could significantly enhance our understanding of the pathogenesis of lung cancer and potentially lead to the development of novel therapeutic approaches. Need for 3D organoids and *in vivo* imaging to study dynamic TME.

## 7 Conclusion

The mechanical properties of tumor ECM play a critical role in tumor therapy and drug resistance. The tumor ECM can directly interact with cell surface receptors, such as integrins, to physically constrain tumor cells or indirectly shield them from apoptotic signals, thereby reducing the efficacy of chemotherapeutic agents. Therefore, modulating the mechanical characteristics of the tumor ECM or interfering with cellular responses to ECM mechanics may represent a promising strategy for promoting tumor cell apoptosis and overcoming drug resistance. Interfering with the mechanotransduction pathways of tumor cells may be one of the effective strategies for cancer treatment: 1) An effective strategy to induce tumor cell apoptosis and inhibit tumor progression by regulating the mechanical properties of tumor cell microenvironment, such as elasticity and viscoelasticity. 2) A method that improves drug permeability, reduces the hardness and pressure of tumor tissues through mechanical loading, thereby promoting drug delivery. 3) The method of regulating the mechanical microenvironment by interfering with the response of tumor cells to mechanical signals.

## Abbreviations

3D, three-dimensional; BMDC, bone marrow-derived dendritic cell; CAFs, cancer-associated fibroblasts; CMs, cell mechanical properties; CS, cyclic stretch; CXCR, 4C-X-C chemokine receptor 4; DDR2, discoidal domain receptor 2; EC, endothelial cells; ECM, extracellular matrix; EMT, epithelial to mesenchymal transition; FA, focal adhesion; FAK, focal adhesion kinase; FET, fibroblasts, human colon cancer cells; LECs, lymphatic endothelial cells; LLC, Lewis lung cancer; LOXL2, lysyl oxidase-like 2; LH2, Lysyl hydroxylase 2; M0, normal macrophages; M2, alternatively activated macrophages; MMP, matrix metalloproteinase; MS, mechanical stress; NF-κB, nuclear transcription factor-κB; NSCLC, non-small cell lung cancer cells; SCC, squamous cell carcinoma; TAFs, tumor-associated fibroblasts; TAM, tumor-associated macrophages; TME, tumor microenvironment; TRPV4, Transient receptor potential vanilloid 4.

## References

[B1] Adapala R. K. Thoppil R. J. Ghosh K. Cappelli H. C. Dudley A. C. Paruchuri S. (2016). Activation of mechanosensitive ion channel trpv4 normalizes tumor vasculature and improves cancer therapy. Oncogene 35 (3), 314–322. 10.1038/onc.2015.83 25867067 PMC4948740

[B2] Adlerz K. M. Aranda-Espinoza H. Hayenga H. N. (2016). Substrate elasticity regulates the behavior of human monocyte-derived macrophages. Eur. Biophysics J. 45 (4), 301–309. 10.1007/s00249-015-1096-8 26613613

[B3] Afik R. Zigmond E. Vugman M. Klepfish M. Shimshoni E. Pasmanik-Chor M. (2016). Tumor macrophages are pivotal constructors of tumor collagenous matrix. J. Exp. Med. 213 (11), 2315–2331. 10.1084/jem.20151193 27697834 PMC5068227

[B4] Alonso-Nocelo M. Raimondo T. M. Vining K. H. López-López R. de la Fuente M. Mooney D. J. (2018). Matrix stiffness and tumor-associated macrophages modulate epithelial to mesenchymal transition of human adenocarcinoma cells. Biofabrication 10 (3), 035004. 10.1088/1758-5090/aaafbc 29595143 PMC5904839

[B5] Aras S. Zaidi M. R. (2017). Tameless traitors: macrophages in cancer progression and metastasis. Br. J. Cancer. 117 (11), 1583–1591. 10.1038/bjc.2017.356 29065107 PMC5729447

[B6] Baday M. Ercal O. Sahan A. Z. Sahan A. Ercal B. Inan H. (2019). Density based characterization of mechanical cues on cancer cells using magnetic levitation. Adv. Healthc. Mat. 8 (10), e1801517. 10.1002/adhm.201801517 30946539

[B7] Bálint L. Jakus Z. (2021). Mechanosensation and mechanotransduction by lymphatic endothelial cells act as important regulators of lymphatic development and function. Int. J. Mol. Sci. 22 (8), 3955. 10.3390/ijms22083955 33921229 PMC8070425

[B8] Ben-Shmuel A. Sabag B. Biber G. Barda-Saad M. (2021). The role of the cytoskeleton in regulating the natural killer cell immune response in health and disease: from signaling dynamics to function. Front. Cell. Dev. Biol. 9, 609532. 10.3389/fcell.2021.609532 33598461 PMC7882700

[B9] Bertero T. Oldham W. M. Grasset E. M. Bourget I. Boulter E. Pisano S. (2019). Tumor-stroma mechanics coordinate amino acid availability to sustain tumor growth and malignancy. Cell Metab. 29 (1), 124–140.e10. 10.1016/j.cmet.2018.09.012 30293773 PMC6432652

[B10] Blakney A. K. Swartzlander M. D. Bryant S. J. (2012). The effects of substrate stiffness on the *in vitro* activation of macrophages and *in vivo* host response to poly(ethylene glycol)-based hydrogels. J. Biomed. Mat. Res. Part A. 100A (6), 1375–1386. 10.1002/jbm.a.34104 22407522 PMC3339197

[B11] Burgess J. K. Harmsen M. C. (2022). Chronic lung diseases: entangled in extracellular matrix. Eur. Respir. Rev. 31 (163), 210202. 10.1183/16000617.0202-2021 35264410 PMC9488575

[B12] Burgstaller G. Oehrle B. Gerckens M. White E. S. Schiller H. B. Eickelberg O. (2017). The instructive extracellular matrix of the lung: basic composition and alterations in chronic lung disease. Eur. Respir. J. 50 (1), 1601805. 10.1183/13993003.01805-2016 28679607

[B13] Butcher D. T. Alliston T. Weaver V. M. (2009). A tense situation: forcing tumour progression. Nat. Rev. Cancer. 9 (2), 108–122. 10.1038/nrc2544 19165226 PMC2649117

[B14] Cappelli H. C. Kanugula A. K. Adapala R. K. Amin V. Sharma P. Midha P. (2019). Mechanosensitive trpv4 channels stabilize ve-cadherin junctions to regulate tumor vascular integrity and metastasis. Cancer Lett. 442, 15–20. 10.1016/j.canlet.2018.07.042 30401632 PMC6924277

[B15] Chen S. Lin J. Yang B. (2014). Modulation of tumor cell stiffness and migration by type iv collagen through direct activation of integrin signaling pathway. Arch. Biochem. Biophys. 555-556, 1–8. 10.1016/j.abb.2014.05.004 24823860

[B16] Chen Y. Terajima M. Yang Y. Sun L. Ahn Y. Pankova D. (2015). Lysyl hydroxylase 2 induces a collagen cross-link switch in tumor stroma. J. Clin. Invest. 125 (3), 1147–1162. 10.1172/JCI74725 25664850 PMC4362236

[B17] Chen H. Cai Y. Chen Q. Li Z. (2020). Multiscale modeling of solid stress and tumor cell invasion in response to dynamic mechanical microenvironment. Biomech. Model. Mechanobiol. 19 (2), 577–590. 10.1007/s10237-019-01231-4 31571083

[B18] Clarke D. L. Carruthers A. M. Mustelin T. Murray L. A. (2013). Matrix regulation of idiopathic pulmonary fibrosis: the role of enzymes. Fibrogenesis and tissue repair 6 (1), 20. 10.1186/1755-1536-6-20 24279676 PMC4176485

[B19] Cross S. E. Jin Y. S. Rao J. Gimzewski J. K. (2007). Nanomechanical analysis of cells from cancer patients. Nat. Nanotechnol. 2 (12), 780–783. 10.1038/nnano.2007.388 18654431

[B20] Deng X. Xiong W. Jiang X. Zhang S. Li Z. Zhou Y. (2020). Lncrna linc00472 regulates cell stiffness and inhibits the migration and invasion of lung adenocarcinoma by binding to ybx1. Cell Death Dis. 11 (11), 945. 10.1038/s41419-020-03147-9 33144579 PMC7609609

[B21] Desai L. P. Chapman K. E. Waters C. M. (2008). Mechanical stretch decreases migration of alveolar epithelial cells through mechanisms involving rac1 and tiam1. Am. J. Physiol.-Lung Cell. Mol. Physiol. 295 (5), L958–L965. 10.1152/ajplung.90218.2008 18805958 PMC2584892

[B22] Dongre A. Weinberg R. A. (2019). New insights into the mechanisms of epithelial-mesenchymal transition and implications for cancer. Nat. Rev. Mol. Cell Biol. 20 (2), 69–84. 10.1038/s41580-018-0080-4 30459476

[B23] Douguet D. Honore E. (2019). Mammalian mechanoelectrical transduction: structure and function of force-gated ion channels. Cell 179 (2), 340–354. 10.1016/j.cell.2019.08.049 31585078

[B24] Doyle A. D. Nazari S. S. Yamada K. M. (2022). Cell–extracellular matrix dynamics. Phys. Biol. 19 (2), 021002. 10.1088/1478-3975/ac4390 34911051 PMC8855216

[B25] Dupont S. Morsut L. Aragona M. Enzo E. Giulitti S. Cordenonsi M. (2011). Role of yap/taz in mechanotransduction. Nature 474 (7350), 179–183. 10.1038/nature10137 21654799

[B26] Emon B. Li Z. Joy M. S. H. Doha U. Kosari F. Saif M. T. A. (2021). A novel method for sensor-based quantification of single/multicellular force dynamics and stiffening in 3d matrices. Sci. Adv. 7 (15), eabf2629. 10.1126/sciadv.abf2629 33837084 PMC8034860

[B27] Friedman D. Simmonds P. Hale A. Bere L. Hodson N. W. White M. R. H. (2021). Natural killer cell immune synapse formation and cytotoxicity are controlled by tension of the target interface. J. Cell Sci. 134 (7), 258570. 10.1242/jcs.258570 33712452 PMC8077183

[B28] Fu X. Kimura Y. Toku Y. Song G. Ju Y. (2023). Metabolic dependency of non-small cell lung cancer cells affected by three-dimensional scaffold and its stiffness. J. Physiol. Biochem. 79 (3), 597–611. 10.1007/s13105-023-00960-6 37213067

[B29] Fujisaki H. Futaki S. (2023). Epithelial–mesenchymal transition induced in cancer cells by adhesion to type i collagen. Int. J. Mol. Sci. 24 (1), 198. 10.3390/ijms24010198 36613638 PMC9820580

[B30] Gladilin E. Ohse S. Boerries M. Busch H. Xu C. Schneider M. (2019). Tgfβ-induced cytoskeletal remodeling mediates elevation of cell stiffness and invasiveness in nsclc. Sci. Rep. 9 (1), 7667. 10.1038/s41598-019-43409-x 31113982 PMC6529472

[B31] Gonçalves I. G. Garcia-Aznar J. M. (2021). Extracellular matrix density regulates the formation of tumour spheroids through cell migration. PLoS Comput. Biol. 17 (2), e1008764. 10.1371/journal.pcbi.1008764 33635856 PMC7968691

[B32] Guo T. He C. Venado A. Zhou Y. (2022). Extracellular matrix stiffness in lung health and disease. Compr. Physiol. 12 (3), 3523–3558. 10.1002/cphy.c210032 35766837 PMC10088466

[B33] Hartmann B. Fleischhauer L. Nicolau M. Jensen T. H. L. Taran F. Clausen-Schaumann H. (2024). Profiling native pulmonary basement membrane stiffness using atomic force microscopy. Nat. Protoc. 19 (5), 1498–1528. 10.1038/s41596-024-00955-7 38429517

[B34] Hsia C. C. W. Hyde D. M. Weibel E. R. (2016). Lung structure and the intrinsic challenges of gas exchange. Compr. Physiol. 6 (2), 827–895. 10.1002/cphy.c150028 27065169 PMC5026132

[B35] Hu B. Xin Y. Hu G. Li K. Tan Y. (2023). Fluid shear stress enhances natural killer cell's cytotoxicity toward circulating tumor cells through nkg2d-mediated mechanosensing. Apl. Bioeng. 7 (3), 036108. 10.1063/5.0156628 37575881 PMC10423075

[B36] Huang Z. Sun Z. Zhang X. Niu K. Wang Y. Zheng J. (2019). Loss of stretch-activated channels, piezos, accelerates non-small cell lung cancer progression and cell migration. Biosci. Rep. 39 (3), BSR20181679. 10.1042/BSR20181679 30745454 PMC6430724

[B37] Hupfer A. Brichkina A. Koeniger A. Keber C. Denkert C. Pfefferle P. (2021). Matrix stiffness drives stromal autophagy and promotes formation of a protumorigenic niche. Proc. Natl. Acad. Sci. 118 (40), e2105367118. 10.1073/pnas.2105367118 34588305 PMC8501848

[B38] Iida K. Sakai R. Yokoyama S. Kobayashi N. Togo S. Yoshikawa H. Y. (2017). Cell softening in malignant progression of human lung cancer cells by activation of receptor tyrosine kinase axl. Sci. Rep. 7 (1), 17770. 10.1038/s41598-017-18120-4 29259259 PMC5736582

[B39] Ikenaga N. Peng Z. Vaid K. A. Liu S. B. Yoshida S. Sverdlov D. Y. (2017). Selective targeting of lysyl oxidase-like 2 (loxl2) suppresses hepatic fibrosis progression and accelerates its reversal. Gut 66 (9), 1697–1708. 10.1136/gutjnl-2016-312473 28073888 PMC5561383

[B40] Ishihara S. Yasuda M. Harada I. Mizutani T. Kawabata K. Haga H. (2013). Substrate stiffness regulates temporary nf-κb activation *via* actomyosin contractions. Exp. Cell Res. 319 (19), 2916–2927. 10.1016/j.yexcr.2013.09.018 24113574

[B41] Ishihara S. Inman D. R. Li W. Ponik S. M. Keely P. J. (2017). Mechano-signal transduction in mesenchymal stem cells induces prosaposin secretion to drive the proliferation of breast cancer cells. Cancer Res. 77 (22), 6179–6189. 10.1158/0008-5472.CAN-17-0569 28972074 PMC5816983

[B42] Ito Y. Correll K. Schiel J. A. Finigan J. H. Prekeris R. Mason R. J. (2014). Lung fibroblasts accelerate wound closure in human alveolar epithelial cells through hepatocyte growth factor/c-met signaling. Am. J. Physiol.-Lung Cell. Mol. Physiol. 307 (1), L94–L105. 10.1152/ajplung.00233.2013 24748602 PMC4080284

[B43] Jeong J. Keum S. Kim D. You E. Ko P. Lee J. (2018). Spindle pole body component 25 homolog expressed by ecm stiffening is required for lung cancer cell proliferation. Biochem. Biophys. Res. Commun. 500 (4), 937–943. 10.1016/j.bbrc.2018.04.205 29709477

[B44] Jiang Y. Zhang H. Wang J. Liu Y. Luo T. Hua H. (2022). Targeting extracellular matrix stiffness and mechanotransducers to improve cancer therapy. J. Hematol. Oncol. 15 (1), 34. 10.1186/s13045-022-01252-0 35331296 PMC8943941

[B45] Kanyo N. Kovacs K. D. Saftics A. Szekacs I. Peter B. Santa-Maria A. R. (2020). Glycocalyx regulates the strength and kinetics of cancer cell adhesion revealed by biophysical models based on high resolution label-free optical data. Sci. Rep. 10 (1), 22422. 10.1038/s41598-020-80033-6 33380731 PMC7773743

[B46] Kessenbrock K. Plaks V. Werb Z. (2010). Matrix metalloproteinases: regulators of the tumor microenvironment. Cell 141 (1), 52–67. 10.1016/j.cell.2010.03.015 20371345 PMC2862057

[B47] Kim D. You E. Jeong J. Ko P. Kim J. Rhee S. (2017). Ddr2 controls the epithelial-mesenchymal-transition-related gene expression *via* c-myb acetylation upon matrix stiffening. Sci. Rep. 7 (1), 6847. 10.1038/s41598-017-07126-7 28754957 PMC5533734

[B48] Kohon A. I. Man K. Mathis K. Webb J. Yang Y. Meckes B. (2023). Nanoparticle targeting of mechanically modulated glycocalyx. bioRxiv, 2023.02.27.529887. 10.1101/2023.02.27.529887 36909503 PMC10002687

[B49] Kraning-Rush C. M. Califano J. P. Reinhart-King C. A. Laird E. G. (2012). Cellular traction stresses increase with increasing metastatic potential. PLoS One 7 (2), e32572. 10.1371/journal.pone.0032572 22389710 PMC3289668

[B50] Kuhn C. McDonald J. A. (1991). The roles of the myofibroblast in idiopathic pulmonary fibrosis. Ultrastructural and immunohistochemical features of sites of active extracellular matrix synthesis. Am. J. Pathol. 138 (5), 1257–1265. 2024710 PMC1886011

[B51] Lech M. Anders H. (2013). Macrophages and fibrosis: how resident and infiltrating mononuclear phagocytes orchestrate all phases of tissue injury and repair. Biochimica Biophysica Acta (BBA) - Mol. Basis Dis. 1832 (7), 989–997. 10.1016/j.bbadis.2012.12.001 23246690

[B52] Lee T. Park J. Y. Lee H. Y. Cho Y. Yoon H. I. Lee J. H. (2014). Lung cancer in patients with idiopathic pulmonary fibrosis: clinical characteristics and impact on survival. Respir. Med. 108 (10), 1549–1555. 10.1016/j.rmed.2014.07.020 25175479

[B53] Li X. Combs J. D. Salaita K. Shu X. (2023). Polarized focal adhesion kinase activity within a focal adhesion during cell migration. Nat. Chem. Biol. 19 (12), 1458–1468. 10.1038/s41589-023-01353-y 37349581 PMC10732478

[B54] Liebman C. McColloch A. Rabiei M. Bowling A. Cho M. (2020). Mechanics of the cell: interaction mechanisms and mechanobiological models. Curr. Top. Membr. 86, 143–184. 10.1016/bs.ctm.2020.09.001 33837692

[B55] Lin B. Fujie H. Yamazaki M. Sakamoto N. (2024). The dual effect of fiber density and matrix stiffness on a549 tumor multicellular migration. Biochem. Biophys. Res. Commun. 741, 151018. 10.1016/j.bbrc.2024.151018 39579534

[B56] Liou Y. R. Torng W. Kao Y. C. Sung K. B. Lee C. H. Kuo P. L. (2014). Substrate stiffness regulates filopodial activities in lung cancer cells. PLoS One 9 (2), e89767. 10.1371/journal.pone.0089767 24587021 PMC3937376

[B57] López-Alonso I. López-Martínez C. Martín-Vicente P. Amado-Rodríguez L. González-López A. Mayordomo-Colunga J. (2022). Mechanical ventilation promotes lung tumour spread by modulation of cholesterol cell content. Eur. Respir. J. 60 (1), 2101470. 10.1183/13993003.01470-2021 34887328

[B58] Mahmoud M. Cancel L. Tarbell J. M. (2021). Matrix stiffness affects glycocalyx expression in cultured endothelial cells. Front. Cell. Dev. Biol. 9, 731666. 10.3389/fcell.2021.731666 34692689 PMC8530223

[B59] Martins M. F. Bairos V. A. (2002). Glycocalyx of lung epithelial cells. Int. Rev. Cytol. 216, 131–173. 10.1016/s0074-7696(02)16005-0 12049207

[B60] McAdams R. M. Mustafa S. B. Shenberger J. S. Dixon P. S. Henson B. M. DiGeronimo R. J. (2006). Cyclic stretch attenuates effects of hyperoxia on cell proliferation and viability in human alveolar epithelial cells. Am. J. Physiol.-Lung Cell. Mol. Physiol. 291 (2), L166–L174. 10.1152/ajplung.00160.2005 16461433 PMC2683386

[B61] McGrail D. J. Kieu Q. M. Iandoli J. A. Dawson M. R. (2015). Actomyosin tension as a determinant of metastatic cancer mechanical tropism. Phys. Biol. 12 (2), 026001. 10.1088/1478-3975/12/2/026001 25706686

[B62] Mierke C. T. (2019). The matrix environmental and cell mechanical properties regulate cell migration and contribute to the invasive phenotype of cancer cells. Rep. Prog. Phys. 82 (6), 064602. 10.1088/1361-6633/ab1628 30947151

[B63] Mina S. G. Huang P. Murray B. T. Mahler G. J. (2017). The role of shear stress and altered tissue properties on endothelial to mesenchymal transformation and tumor-endothelial cell interaction. Biomicrofluidics 11 (4), 044104. 10.1063/1.4991738 28798857 PMC5533495

[B64] Mordechay L. Le Saux G. Edri A. Hadad U. Porgador A. Schvartzman M. (2021). Mechanical regulation of the cytotoxic activity of natural killer cells. ACS Biomater. Sci. Eng. 7 (1), 122–132. 10.1021/acsbiomaterials.0c01121 33455204

[B65] Navab R. Strumpf D. To C. Pasko E. Kim K. S. Park C. J. (2016). Integrin α11β1 regulates cancer stromal stiffness and promotes tumorigenicity and metastasis in non-small cell lung cancer. Oncogene 35 (15), 1899–1908. 10.1038/onc.2015.254 26148229 PMC4833874

[B66] Neumann P. Jaé N. Knau A. Glaser S. F. Fouani Y. Rossbach O. (2018). The lncrna gata6-as epigenetically regulates endothelial gene expression *via* interaction with loxl2. Nat. Commun. 9 (1), 237. 10.1038/s41467-017-02431-1 29339785 PMC5770451

[B67] Nguyen E. V. Pereira B. A. Lawrence M. G. Ma X. Rebello R. J. Chan H. (2019). Proteomic profiling of human prostate cancer-associated fibroblasts (Caf) reveals loxl2-dependent regulation of the tumor microenvironment. Mol. and Cell. Proteomics 18 (7), 1410–1427. 10.1074/mcp.RA119.001496 31061140 PMC6601211

[B68] Novak C. Ballinger M. N. Ghadiali S. (2021). Mechanobiology of pulmonary diseases: a review of engineering tools to understand lung mechanotransduction. J. Biomechanical Eng. 143 (11), 110801. 10.1115/1.4051118 33973005 PMC8299813

[B69] Oliver G. Kipnis J. Randolph G. J. Harvey N. L. (2020). The lymphatic vasculature in the 21(st) century: novel functional roles in homeostasis and disease. Cell 182 (2), 270–296. 10.1016/j.cell.2020.06.039 32707093 PMC7392116

[B70] Pankova D. Chen Y. Terajima M. Schliekelman M. J. Baird B. N. Fahrenholtz M. (2016). Cancer-associated fibroblasts induce a collagen cross-link switch in tumor stroma. Mol. Cancer Res. 14 (3), 287–295. 10.1158/1541-7786.MCR-15-0307 26631572 PMC4794404

[B71] Phan T. K. T. Shahbazzadeh F. Kihara T. (2020). Alpha-mangostin reduces mechanical stiffness of various cells. Hum. Cell. 33 (2), 347–355. 10.1007/s13577-020-00330-0 32078151

[B72] Planas-Paz L. Lammert E. (2014). Mechanosensing in developing lymphatic vessels. Adv. Anat. Embryol. Cell. Biol. 214, 23–40. 10.1007/978-3-7091-1646-3_3 24276884

[B73] Previtera M. L. Sengupta A. (2015). Substrate stiffness regulates proinflammatory mediator production through tlr4 activity in macrophages. PLoS One 10 (12), e0145813. 10.1371/journal.pone.0145813 26710072 PMC4692401

[B74] Puig M. Lugo R. Gabasa M. Giménez A. Velásquez A. Galgoczy R. (2015). Matrix stiffening and β1 integrin drive subtype-specific fibroblast accumulation in lung cancer. Mol. Cancer Res. 13 (1), 161–173. 10.1158/1541-7786.MCR-14-0155 25280968

[B75] Quante M. Tu S. P. Tomita H. Gonda T. Wang S. S. W. Takashi S. (2011). Bone marrow-derived myofibroblasts contribute to the mesenchymal stem cell niche and promote tumor growth. Cancer Cell 19 (2), 257–272. 10.1016/j.ccr.2011.01.020 21316604 PMC3060401

[B76] Rahir G. Moser M. (2012). Tumor microenvironment and lymphocyte infiltration. Cancer Immunol. Immunother. 61 (6), 751–759. 10.1007/s00262-012-1253-1 22488275 PMC11028584

[B77] Saijo A. Goto H. Nakano M. Mitsuhashi A. Aono Y. Hanibuchi M. (2018). Bone marrow-derived fibrocytes promote stem cell-like properties of lung cancer cells. Cancer Lett. 421, 17–27. 10.1016/j.canlet.2018.02.016 29448000

[B78] Sakpakdeejaroen I. Muanrit P. Panthong S. Ruangnoo S. (2022). Alpha-mangostin-loaded transferrin-conjugated lipid-polymer hybrid nanoparticles: development and characterization for tumor-targeted delivery. Sci. World J. 2022, 9217268–10. 10.1155/2022/9217268 36081606 PMC9448606

[B79] Shikata Y. Rios A. Kawkitinarong K. Depaola N. Garcia J. Birukov K. (2005). Differential effects of shear stress and cyclic stretch on focal adhesion remodeling, site-specific fak phosphorylation, and small gtpases in human lung endothelial cells. Exp. Cell Res. 304 (1), 40–49. 10.1016/j.yexcr.2004.11.001 15707572

[B80] Shu J. Deng H. Zhang Y. Wu F. He J. (2024). Cancer cell response to extrinsic and intrinsic mechanical cue: opportunities for tumor apoptosis strategies. Regen. Biomater. 11, rbae016. 10.1093/rb/rbae016 38476678 PMC10932484

[B81] Shukla V. C. Higuita-Castro N. Nana-Sinkam P. Ghadiali S. N. (2016). Substrate stiffness modulates lung cancer cell migration but not epithelial to mesenchymal transition. J. Biomed. Mater. Res. Part A 104 (5), 1182–1193. 10.1002/jbm.a.35655 26779779

[B82] Sicard D. Fredenburgh L. E. Tschumperlin D. J. (2017). Measured pulmonary arterial tissue stiffness is highly sensitive to afm indenter dimensions. J. Mech. Behav. Biomed. Mat. 74, 118–127. 10.1016/j.jmbbm.2017.05.039 28595103 PMC5582007

[B83] Sneider A. Liu Y. Starich B. Du W. Nair P. R. Marar C. (2024). Small extracellular vesicles promote stiffness-mediated metastasis. Cancer Res. Commun. 4 (5), 1240–1252. 10.1158/2767-9764.CRC-23-0431 38630893 PMC11080964

[B84] Sohrabi Kashani A. Larocque K. Piekny A. Packirisamy M. (2022). Gold nano-bio-interaction to modulate mechanobiological responses for cancer therapy applications. ACS Appl. Bio Mat. 5 (8), 3741–3752. 10.1021/acsabm.2c00230 35839330

[B85] Sousa S. Brion R. Lintunen M. Kronqvist P. Sandholm J. Mönkkönen J. (2015). Human breast cancer cells educate macrophages toward the m2 activation status. Breast Cancer Res. 17 (1), 101. 10.1186/s13058-015-0621-0 26243145 PMC4531540

[B86] Suki B. Bates J. Bartolak-Suki E. (2022). Remodeling of the aged and emphysematous lungs: roles of microenvironmental cues. Compr. Physiol. 12 (3), 3559–3574. 10.1002/cphy.c210033 35766835 PMC11470990

[B87] Sun B. Qu R. Fan T. Yang Y. Jiang X. Khan A. U. (2021). Actin polymerization state regulates osteogenic differentiation in human adipose-derived stem cells. Cell. Mol. Biol. Lett. 26 (1), 15. 10.1186/s11658-021-00259-8 33858321 PMC8048231

[B88] Thoelking G. Reiss B. Wegener J. Oberleithner H. Pavenstaedt H. Riethmuller C. (2010). Nanotopography follows force in tgf-beta1 stimulated epithelium. Nanotechnology 21 (26), 265102. 10.1088/0957-4484/21/26/265102 20522928

[B89] Thoppil R. J. Cappelli H. C. Adapala R. K. Kanugula A. K. Paruchuri S. Thodeti C. K. (2016). Trpv4 channels regulate tumor angiogenesis *via* modulation of rho/rho kinase pathway. Oncotarget 7 (18), 25849–25861. 10.18632/oncotarget.8405 27029071 PMC5041949

[B90] Tian H. Shi H. Yu J. Ge S. Ruan J. (2022). Biophysics role and biomimetic culture systems of ecm stiffness in cancer emt. Glob. Chall. 6 (6), 2100094. 10.1002/gch2.202100094 35712024 PMC9189138

[B91] Tilghman R. W. Cowan C. R. Mih J. D. Koryakina Y. Gioeli D. Slack-Davis J. K. (2010). Matrix rigidity regulates cancer cell growth and cellular phenotype. PLoS One 5 (9), e12905. 10.1371/journal.pone.0012905 20886123 PMC2944843

[B92] Torrino S. Roustan F. R. Kaminski L. Bertero T. Pisano S. Ambrosetti D. (2019). Ubtd1 is a mechano‐regulator controlling cancer aggressiveness. EMBO Rep. 20 (4), e46570. 10.15252/embr.201846570 30804013 PMC6446246

[B93] Torrino S. Grasset E. M. Audebert S. Belhadj I. Lacoux C. Haynes M. (2021). Mechano-induced cell metabolism promotes microtubule glutamylation to force metastasis. Cell Metab. 33 (7), 1342–1357.e10. 10.1016/j.cmet.2021.05.009 34102109

[B94] Uhlen M. Fagerberg L. Hallstrom B. M. Lindskog C. Oksvold P. Mardinoglu A. (2010). Towards a knowledge-based human protein atlas. Nat. Biotechnol. 28 (12), 1248–1250. 10.1038/nbt1210-1248 21139605

[B95] Uhlen M. Fagerberg L. Hallstrom B. M. Lindskog C. Oksvold P. Mardinoglu A. (2015). Proteomics. Tissue-based map of the human proteome. Science 347 (6220), 1260419. 10.1126/science.1260419 25613900

[B96] Ulldemolins A. Narciso M. Sanz-Fraile H. Otero J. Farré R. Gavara N. (2024). Effects of aging on the biomechanical properties of the lung extracellular matrix: dependence on tissular stretch. Front. Cell. Dev. Biol. 12, 1381470. 10.3389/fcell.2024.1381470 38645411 PMC11026642

[B97] van Deventer H. W. Palmieri D. A. Wu Q. P. McCook E. C. Serody J. S. (2013). Circulating fibrocytes prepare the lung for cancer metastasis by recruiting ly-6c+ monocytes *via* ccl2. J. Immunol. (1950) 190 (9), 4861–4867. 10.4049/jimmunol.1202857 23536638 PMC3740355

[B98] Wang Y. Wang W. Li Z. Hao S. Wang B. (2016). A novel perspective on neuron study: damaging and promoting effects in different neurons induced by mechanical stress. Biomech. Model. Mechanobiol. 15 (5), 1019–1027. 10.1007/s10237-015-0743-4 26591000

[B99] Wang W. Lollis E. M. Bordeleau F. Reinhart-King C. A. (2019a). Matrix stiffness regulates vascular integrity through focal adhesion kinase activity. FASEB J. 33 (1), 1199–1208. 10.1096/fj.201800841R 30102569 PMC6355084

[B100] Wang Y. X. Wang D. Y. Guo Y. C. Guo J. (2019b). Zyxin: a mechanotransductor to regulate gene expression. Eur. Rev. Med. Pharmacol. Sci. 23 (1), 413–425. 10.26355/eurrev_201901_16790 30657586

[B101] Wang W. Hsu C. Huang H. Juan H. (2020). Quantitative phosphoproteomics reveals cell alignment and mitochondrial length change under cyclic stretching in lung cells. Int. J. Mol. Sci. 21 (11), 4074. 10.3390/ijms21114074 32517296 PMC7312583

[B102] Waters C. M. Roan E. Navajas D. (2012). Mechanobiology in lung epithelial cells: measurements, perturbations, and responses Compr. Physiol., 2 (1), 1–29. 10.1002/cphy.c100090 23728969 PMC4457445

[B103] Wei Y. Kim T. J. Peng D. H. Duan D. Gibbons D. L. Yamauchi M. (2017). Fibroblast-specific inhibition of tgf-β1 signaling attenuates lung and tumor fibrosis. J. Clin. Invest. 127 (10), 3675–3688. 10.1172/JCI94624 28872461 PMC5617667

[B104] Wen B. Xu L. Li E. (2020). Loxl2 in cancer: regulation, downstream effectors and novel roles. Biochimica Biophysica Acta (BBA) - Rev. Cancer 1874 (2), 188435. 10.1016/j.bbcan.2020.188435 32976981

[B105] Wohl I. Sajman J. Sherman E. (2023). Cell surface vibrations distinguish malignant from benign cells. Cells 12 (14), 1901. 10.3390/cells12141901 37508565 PMC10378100

[B106] Wu T. H. Chou Y. W. Chiu P. H. Tang M. J. Hu C. W. Yeh M. L. (2014). Validation of the effects of TGF-β1 on tumor recurrence and prognosis through tumor retrieval and cell mechanical properties. Cancer Cell Int. 14 (1), 20. 10.1186/1475-2867-14-20 24581230 PMC3973896

[B107] Wu S. Zheng Q. Xing X. Dong Y. Wang Y. You Y. (2018). Matrix stiffness-upregulated loxl2 promotes fibronectin production, mmp9 and cxcl12 expression and bmdcs recruitment to assist pre-metastatic niche formation. J. Exp. Clin. Cancer Res. 37 (1), 99. 10.1186/s13046-018-0761-z 29728125 PMC5935912

[B108] Wu J. Zhang Q. Yang Z. Xu Y. Liu X. Wang X. (2024). Cd248 ‐expressing cancer‐associated fibroblasts induce non‐small cell lung cancer metastasis *via* hippo pathway‐mediated extracellular matrix stiffness. J. Cell. Mol. Med. 28 (16), e70025. 10.1111/jcmm.70025 39164826 PMC11335579

[B109] Yanamandra A. K. Zhang J. Montalvo G. Zhou X. Biedenweg D. Zhao R. (2024). Piezo1‐mediated mechanosensing governs nk‐cell killing efficiency and infiltration in three‐dimensional matrices. Eur. J. Immunol. 54 (3), 2350693. 10.1002/eji.202350693 38279603

[B110] Yang N. Chen T. Wang L. Liu R. Niu Y. Sun L. (2020). Cxcr4 mediates matrix stiffness-induced downregulation of ubtd1 driving hepatocellular carcinoma progression *via* Yap signaling pathway. Theranostics 10 (13), 5790–5801. 10.7150/thno.44789 32483419 PMC7255012

[B111] Zeltz C. Primac I. Erusappan P. Alam J. Noel A. Gullberg D. (2020). Cancer-associated fibroblasts in desmoplastic tumors: emerging role of integrins. Semin. Cancer. Biol. 62, 166–181. 10.1016/j.semcancer.2019.08.004 31415910

[B112] Zhang K. Rekhter M. D. Gordon D. Phan S. H. (1994). Myofibroblasts and their role in lung collagen gene expression during pulmonary fibrosis. A combined immunohistochemical and *in situ* hybridization study. Am. J. Pathol. 145 (1), 114–125. 7518191 PMC1887314

[B113] Zhang H. Maric I. DiPrima M. J. Khan J. Orentas R. J. Kaplan R. N. (2013). Fibrocytes represent a novel mdsc subset circulating in patients with metastatic cancer. Blood 122 (7), 1105–1113. 10.1182/blood-2012-08-449413 23757729 PMC3744987

[B114] Zhang X. Wang X. Ni S. Qin W. Zhao L. Hua R. (2015). Ubtd1 induces cellular senescence through an ubtd1-mdm2/p53 positive feedback loop. J. Pathology 235 (4), 656–667. 10.1002/path.4478 25382750

[B115] Zhang C. Zhu M. Wang H. Wen J. Huang Z. Chen S. (2021). Loxl2 attenuates osteoarthritis through inactivating integrin/fak signaling. Sci. Rep. 11 (1), 17020. 10.1038/s41598-021-96348-x 34426599 PMC8382747

[B116] Zhang Y. Fu Q. Sun W. Yue Q. He P. Niu D. (2025). Mechanical forces in the tumor microenvironment: roles, pathways, and therapeutic approaches. J. Transl. Med. 23 (1), 313. 10.1186/s12967-025-06306-8 40075523 PMC11899831

[B117] Zhou X. Zhao R. Schwarz K. Mangeat M. Schwarz E. C. Hamed M. (2017). Bystander cells enhance nk cytotoxic efficiency by reducing search time. Sci. Rep. 7 (1), 44357. 10.1038/srep44357 28287155 PMC5347013

[B118] Zhou H. Wang M. Zhang Y. Su Q. Xie Z. Chen X. (2022). Functions and clinical significance of mechanical tumor microenvironment: cancer cell sensing, mechanobiology and metastasis. Cancer Commun. 42 (5), 374–400. 10.1002/cac2.12294 35470988 PMC9118059

[B119] Zwaans B. M. M. Grobbel M. Carabulea A. L. Lamb L. E. Roccabianca S. (2022). Increased extracellular matrix stiffness accompanies compromised bladder function in a murine model of radiation cystitis. Acta Biomater. 144, 221–229. 10.1016/j.actbio.2022.03.017 35301146 PMC9100859

